# Characterization of the Viral Microbiome in Patients with Severe Lower Respiratory Tract Infections, Using Metagenomic Sequencing

**DOI:** 10.1371/journal.pone.0030875

**Published:** 2012-02-15

**Authors:** Fredrik Lysholm, Anna Wetterbom, Cecilia Lindau, Hamid Darban, Annelie Bjerkner, Kristina Fahlander, A. Michael Lindberg, Bengt Persson, Tobias Allander, Björn Andersson

**Affiliations:** 1 IFM Bioinformatics and Swedish e-Science Research Centre (SeRC), Linköping University, Linköping, Sweden; 2 Department of Cell and Molecular Biology, Science for Life Laboratory, Karolinska Institutet, Stockholm, Sweden; 3 Karolinska Institutet, Department of Microbiology, Tumor- and Cell Biology, Laboratory for Clinical Microbiology, Karolinska University Hospital, Stockholm, Sweden; 4 School of Natural Sciences, Linnaeus University, Kalmar, Sweden; Baylor College of Medicine, United States of America

## Abstract

The human respiratory tract is heavily exposed to microorganisms. Viral respiratory tract pathogens, like RSV, influenza and rhinoviruses cause major morbidity and mortality from respiratory tract disease. Furthermore, as viruses have limited means of transmission, viruses that cause pathogenicity in other tissues may be transmitted through the respiratory tract. It is therefore important to chart the human virome in this compartment. We have studied nasopharyngeal aspirate samples submitted to the Karolinska University Laboratory, Stockholm, Sweden from March 2004 to May 2005 for diagnosis of respiratory tract infections. We have used a metagenomic sequencing strategy to characterize viruses, as this provides the most unbiased view of the samples. Virus enrichment followed by 454 sequencing resulted in totally 703,790 reads and 110,931 of these were found to be of viral origin by using an automated classification pipeline. The snapshot of the respiratory tract virome of these 210 patients revealed 39 species and many more strains of viruses. Most of the viral sequences were classified into one of three major families; *Paramyxoviridae*, *Picornaviridae* or *Orthomyxoviridae*. The study also identified one novel type of Rhinovirus C, and identified a number of previously undescribed viral genetic fragments of unknown origin.

## Introduction

Respiratory tract infections account for great morbidity and mortality in the human population and caused almost 4 million deaths in 2008 [1]. A large proportion of these infections have viral etiology, in particular in children. While previous studies have identified a number of viral etiologic agents, such as rhinovirus, coronavirus, influenzavirus, parainfluenzavirus, respiratory syncytial virus and adenovirus, approximately 30% of all presumed viral cases fail diagnostic tests for these agents [2]. Thus, the tests are either inefficient or the causative agent is unrelated to any of the known viruses associated with respiratory infections. In fact, since 2001, several previously undescribed viruses have been identified by analysis of the human respiratory tract, including metapneumovirus [3], severe acute respiratory syndrome (SARS) [4] and human bocavirus [5].

Viruses have limited means of transmission between organisms, but the respiratory tract is one important route. Many viruses that are primarily associated with non-respiratory infections, for example, herpes viruses, enteroviruses and parvovirus B19 [6], [7], are still transmitted through the respiratory tract. Therefore, the respiratory tract is an excellent starting point for an in-depth characterization of the human virome and to identify novel human viruses.

In recent years, viral metagenomics has become an established method both for finding novel viruses and for detecting the presence of known viruses in new environments [5], [8], [9], [10], [11]. We have sequenced and characterized the virome, in respiratory tract secretions from hospitalized patients, mainly infants and children, with severe lower respiratory tract infections. While many pathogens of the respiratory tract is of bacterial origin, due to chemical enrichment for virus, the bacterial sequences found in this study are likely biased and not representative for these patients. Even so, we have provided a crude characterization of the bacterial content found in these samples along with contigs of other origin. We confirmed that the lower respiratory tract was a milieu that was rich in viruses, in these patients. Many known pathogens were identified, but we also found unexpected virus families as well as one novel rhinovirus C type.

## Results

### Data analysis pipeline

**Figure 1:**
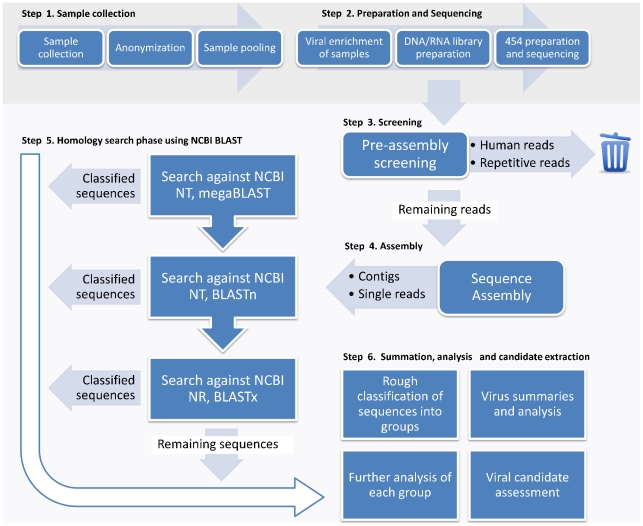
A flow-chart describing the classification pipeline. A flow-chart describing the entire process from sample collection through the various data-analysis steps. Step 1 and 2 illustrate sample collection, preparation and sequencing while step 3 through 6 illustrate *in silico* efforts.

In analysis of 210 pool patient samples we have developed an in-house metagenomic sequence analysis pipeline for screening the sequencing reads, assembling the reads into contigs and finally to carry out extensive homology searches to classify contigs and singletons (outlined in Figure 1). The analysis pipeline was comprised of a series of Shell, Perl, Python scripts and C++ programs, and is available for download at http://www.ifm.liu.se/bioinfo/.

#### Pre-processing of data and de novo genome assembly

Metagenomic data typically include sequences from a multitude of species and strains. In order to reduce the complexity of the data, putative human sequences and repetitive sequences were removed before the assembly process. This pre-processing step improved the accuracy of the assembled genomes; see Methods for a complete description of the analysis pipeline. The pre-assembly screening removed almost a 60% of the 454 reads (see Table S1) and the RNA-derived library contained fewer human and repetitive reads as compared to the DNA-derived library. This likely reflected the nature of the input genetic material, where more human and bacterial genomic DNA was carried through to the DNA pool, whereas cellular RNA contributed to a lesser extent to the RNA pools, possibly due to lower stability of cellular RNA compared to viral RNA. All remaining reads were subsequently used for de novo assembly with the MIRA assembler [12]. Assembling the sequencing reads into longer contigs significantly increased the accuracy by which sequences could be classified. Approximately 35% of the reads did not assemble into contigs but in the following discussion these singeltons will also be included in the term contig. Assembly details and statistics are described in Table S2.

#### Inferring homology for the assembled contigs

**Figure 2:**
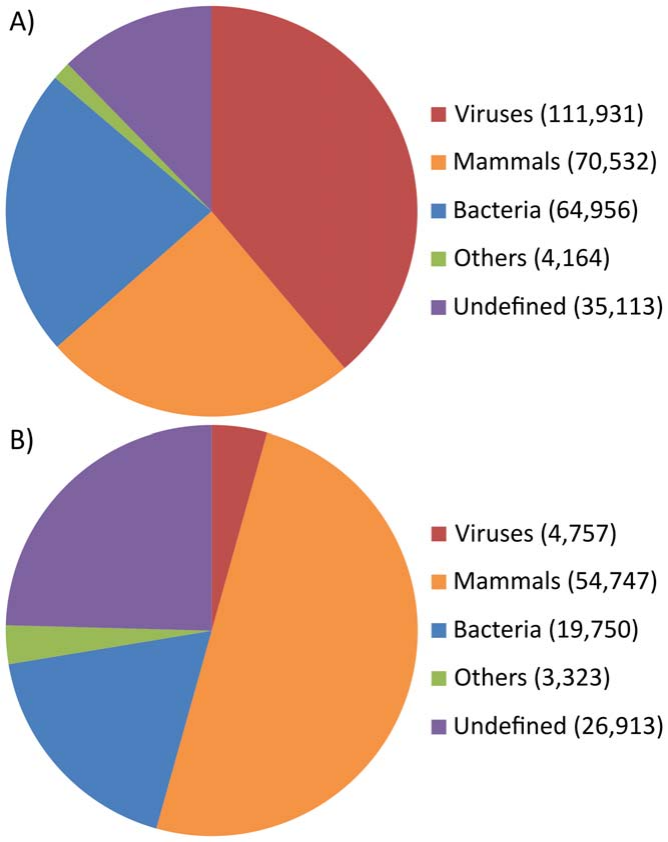
Major classification of sample content. The contigs defined by relative position-wise homology (see Methods) and split into Viruses, Bacteria, Mammals, Others or none of the four where in the cases where it could not clearly be determined (Undefined). The numbers are the derived number of reads (panel A) and the number of sequences (panel B).

In the final homology search phase, each contig was searched against the NCBI nt (minimally non-redundant nucleotide) and nr (non-redundant protein) databases [13]. Local alignments were produced using BLAST [14] and the highest scoring hit was assigned as the closest homolog. Based on the inferred homology, the sequences were divided into the following categories: viruses, bacteria, mammals, others and undefined (see Figure 2). The ‘undefined’ category included sequences that lacked a known homolog, or for which almost equally ‘close’ homologs were found in more than one category; see Materials and Methods for a detailed description of category assignment. All sequences that were reliably classified as non-human were submitted to Genbank, as a GenomeProject, ID 64629.

### Microbiome characterization

#### Quantification of the sample content

For the purpose of characterizing the sample content we have compared the number of reads, derived from the assembled contigs, rather than comparing the number of contigs, since the amount of sequenced reads is more directly correlated with the amount of DNA in the original sample. The reason for this is the variation in copy numbers and sizes of the various genomes that were present in the original samples. This was evident from Figure 2 for example, where almost 40% of the sequence reads were of viral origin but after assembly only 4% of the assembled contigs were classified as viral.

#### Contigs showing non-viral homology

The largest non-viral portion of the libraries, even after the initial removal of most human sequences, consisted of contigs of putative mammalian origin (Figure 2A) and we expected that these were almost exclusively human. Based on the closest homolog, 98% of the mammalian sequences were of human origin. Except for the mitochondrion, which had higher coverage, the distribution across all human chromosomes was even, considering the size of each chromosome (Table S3). For the remaining 2% of the mammalian sequences, the closest homolog originated from other primates or other mammals, for example rodents. A possible explanation for this is that the human homolog has in some cases not yet been reported in public databases. Another possibility is the introduction of small amounts of animal DNA through the molecular biology reagents used in the library construction process.

**Figure 3:**
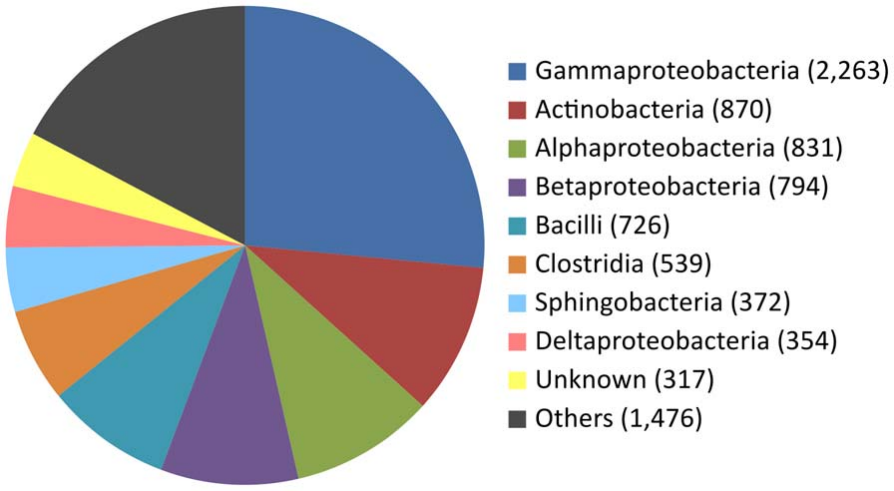
Class distribution of bacterial content. The bacterial part of the sample where sequences were defined by closest homolog, and split into classes. The numbers are the derived number of reads.

Bacterial sequences made up the second largest non-viral portion of the data set, 23% of the sequence reads (Figure 2A). The bacterial contigs were split further into classes, as defined by their closest homolog (summarized in Figure 3). To gain further insight, the sample was split into putative species by using the closest homolog. Bacterial species for which more than 30 sequences were found are shown in Table 1. These included *Haemophilus influenzae*, *Streptococcus pneumoniae* and *Moraxella catarrhalis*, which are known to frequently colonize the nasopharynx of infants and children and are also common pathogens in the respiratory tract [15]. However, since chemical and physical purification for virus was employed prior to sequencing all bacterial findings are likely to be biased and are not representative for these samples. To further investigate this likely bias the bacterial content was analyzed further in regard to ribosomal RNA (rRNA) or genomic origin, see Table 2. As seen in this table, 61% of the leakage of bacterial sequences into the RNA pool was rRNA while rRNA was only 5% of the DNA pool.

**Table 1:** Bacteria species found in the sample.

Bacteria species	Contigs	Reads (derived from contigs)
Delftia acidovorans	1,194	18,388
Pediococcus acidilactici	279	4,668
Comamonas testosterone	853	3,944
**Haemophilus influenzae**	**205**	**1,951**
Acinetobacter baumannii	1,097	1,486
Pseudomonas fluorescens	450	1,399
Lactobacillus sakei	96	1,312
Delftia sp lp-1	86	1,000
Staphylococcus epidermidis	64	876
Acinetobacter calcoaceticus	43	664
**Streptococcus pneumoniae**	**184**	**629**
Acinetobacter sp adp1	280	571
Methylotenera mobilis	47	481
Escherichia coli	78	353
Bacillus subtilis	288	346
Chitinophaga pinensis	330	338
Delftia sp lp2mm	113	329
Bacillus cereus	214	327
Flavobacterium johnsoniae	316	326
Curvibacter putative symbiont of hydra magnipapillata	109	290
Granulicatella adiacens	256	272
Flavobacterium psychrophilum	256	261
Pedobacter heparinus	204	223
Diaphorobacter sp tpsy	76	206
**Moraxella catarrhalis**	**50**	**164**
Thiobacillus denitrificans	38	144
Acinetobacter sp atcc 27244	118	142
Acinetobacter junii	110	140
Spirosoma lingual	121	124

The top thirty contigs split by bacteria species defined by closest homolog and sorted by descending total number derived reads and contigs. The table only list contigs present in the bacteria category (see Methods for a complete description of requirement for category assignment).

**Table 2:** Genomic origin of bacterial contigs.

	No. Sequences	No. of reads	RNA reads	DNA reads
**Genomic**	16,493	31,764	20,785	10,979
**rRNA**	3,257	33,192	32,634	558
**16S**	2,414	26,293	25,981	312
**23S**	1,569	19,041	18,755	286
**Total**	**19,750**	**64,956**	**53,419**	**11,537**

The genomic origin of bacterial contigs as well as derived reads, DNA and RNA reads) based on closest homologs.

A small portion (1.4%) of the contigs (Figure 2A) was classified as being from other organisms, besides bacteria, mammals or animal viruses. A further split into NCBI taxonomy divisions [13] defined by closest homolog of these sequences was performed, see Table S4. The sequences included hits to various NCBI divisions of life including phages and fungi. Considering all sequences, all NCBI divisions turned out to be represented by at least a few sequences.

Finally, approximately 12% of the sequences could not be classified, since no homolog was found (e-value>10^−3^) or there were contradicting database hits. In Figure 2A this category is referred to as ‘undefined’. The ambiguous part of ‘undefined’ (23974/35113) was split into taxonomy divisions, using the closest homolog (as if non-ambiguous). The resulting, uncertain, major divisions were environmental samples, primate and bacteria (see Table S5).

#### Contigs showing viral homology

**Figure 4:**
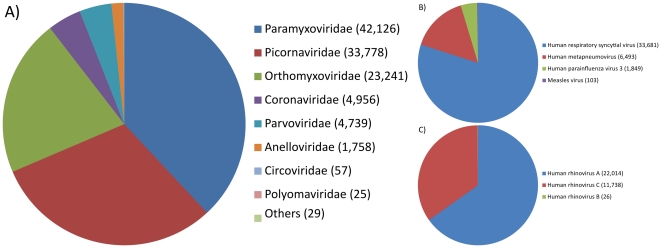
An overview of the viral part of the sample. The virus part of the sample, where sequences were defined by closest homolog, split into families (panel A), species found in the *Paramyxoviridae* family (panel B) and species found in the *Picornaviridae* family (panel C). The numbers are the derived number of reads and the family and species designations were manually curated, only alignments with an e-value<1e-5 considered.

Viruses made up 39% (n = 110,931) of the reads derived from contigs and thus represented the largest portion of the samples after human and repetitive sequences were removed (Figure 2A). Contigs found to be of viral origin were further divided into virus families based on their closest homolog (Figure 4A) and Table 3 shows the complete list of the identified viruses. To provide a more reliable list of families and species the list was manually curated to address yet unclassified strains which dilute the species designation. Furthermore, alignments assigned an e-value≥1e-5, 184 sequences, could not be reliably classified. The initial overview showed, as expected, that the main virus species that have previously been associated with LRTI (lower respiratory tract infections) in children were present in these samples. For example, families such as Paramyxoviridae, Orthomyxoviridae and Picornaviridae constituted a large proportion of the viral sequences. Within these families several species/types were found and especially within the Picornaviridae family many divergent sequences were found, suggesting the presence of potential new types (see below). Furthermore, we also identified human bocavirus and a multitude of less common viruses, such as measles virus, circovirus and human picobirnavirus. Human picobirnavirus has not previously been described in nasopharyngeal aspirates.

**Table 3:** Virus species found in the sample.

Virus species	Contigs	Reads (derived from contigs)
Human respiratory syncytial virus	1,453	33,681
Influenza A virus	746	22,325
Human rhinovirus A	585	22,014
Human rhinovirus C	331	11,738
Human metapneumovirus	416	6,493
Human bocavirus	154	4,725
Human coronavirus HKU1	186	4,098
Human parainfluenza virus 3	88	1,849
Torque teno midi virus & TTV-like mini virus	159	1,514
Human coronavirus OC43 (betacoronavirus 1)	156	860
Influenza B virus	59	530
Influenza C virus	39	386
Small anellovirus	28	192
Measles virus	52	103
Circovirus	11	55
Torque teno virus	45	50
Human rhinovirus B	20	26
KI polyomavirus	12	25
Feline panleukopenia virus	1	8
Human picobirnavirus	1	6
Blattella germanica densovirus	4	4
Penicillium chrysogenum virus	4	4
African swine fever virus	2	2
White spot syndrome virus 1	2	2
Paramecium bursaria Chlorella virus A1	2	2
Banana bunchy top virus	2	2
Human adenovirus C	2	2
Acanthamoeba polyphaga mimivirus	2	2
Cryphonectria nitschkei chrysovirus 1	2	2

The contigs split by virus species defined through closest homolog and sorted by the descending total number of derived reads and contigs. The species list have been manually curated and grouped in cases where yet unclassified strains dilute the species designation. Species producing a single read have also been removed. Furthermore, all alignments with an e-value at or above 1e-5 were ignored (this excluded 184 sequences, for which no species designation is provided).

Amongst the species found at high titer, such as HRV (human rhinovirus) from *Picornaviridae*, there was clear evidence of strains that were represented only by a few sequence reads and we therefore concluded that all viral sequences were not covered by the four 454 sequencing runs. Even so, the analysis clearly showed that a number of hitherto unknown potential pathogens could be and have been discovered using our strategy [5], [16].

### Three common virus families

Common causes for severe lower respiratory tract infections in children, and to some extent in adults, are human respiratory syncytial virus (hRSV), human metapneumovirus (hMPV), human parainfluenza virus (hPIV), influenza virus, and human rhinovirus [17], [18]. These viruses belong to three families of RNA viruses; *Paramyxoviridae* (hRSV, hMPV, and hPIV), *Orthomyxoviridae* (influenza virus) and *Picornaviridae* (HRV). These three families together comprised almost 90% of the viral contigs in the samples (Figure 4A).

#### Paramyxoviridae

The most abundant virus family in these samples was Paramyxoviridae, which accounted for 38% of the viral content (Figure 4A). The sequences from this family included 80% human respiratory syncytial virus (hRSV) related reads and 15% human metapneumovirus (hMPV), see Figure 4B. This confirmed previous studies of children with severe lower respiratory tract infections, where both hRSV and hMPV were common [17]. Approximately half of the hRSV homologs were contigs of more than one read and the nucleotide identity to known strains of hRSV varied from 82–100%, for alignments covering at least 100 bp. The contigs homologous to hRSV of identity bellow 90% could potentially be of new types of hRSV and are spread amongst several genes including; L (large), N (nucleoprotein) and G (glycoprotein). However, all of these are single read contigs where read quality could affect the results and without longer contigs it is impossible to investigate the possibility of new hRSV types. Similarly, approximately half of the hMPV homologs were also contigs of several reads and the nucleotide identity to known strains varied from 88–100%.

The RNA-derived library contained 52 contigs (103 reads, see Figure 4B) that were homologous to the measles virus. The nucleotide identity towards known sequences varied from 91% to 100% (for local alignments covering at least 100 bp). Due to high vaccination coverage, measles outbreaks are rare in Sweden. However, measles cases were reported during the sampling time period, and the measles sequences were most likely derived from one or more actual measles cases. In order to verify this, the sequences were compared to the MMR vaccine strain (AF266290.1). 34 out of 42 contigs (only accounting for contigs which had an alignment towards a database sequence that covered at least 100 bp) showed lower nucleotide identity compared to the vaccine strain than to other measles database entries. Six out of these 34 showed an alignment identity difference of more than 3%. All these six were alignments of more than 200 bp, among them the longest contig of 402 bp, which showed 90.7% identity to the vaccine strain while having 95% identity towards the T11wild strain (AB481087.1). This indicated that the measles virus sequences that were found in these samples are derived from wild-type measles rather than exposure to the MMR vaccine.

#### Picornaviridae

The second most abundant virus family was Picornaviridae, which accounted for 31% of the sequence reads (Figure 4A). The family was split further into rhinovirus A (65%), rhinovirus B (0.1%) and rhinovirus C (35%), see Figure 4B. The rhinovirus A and B homologs showed more than 90% residue identity to known strains while the rhinovirus C homologs showed more divergence.

**Figure 5:**
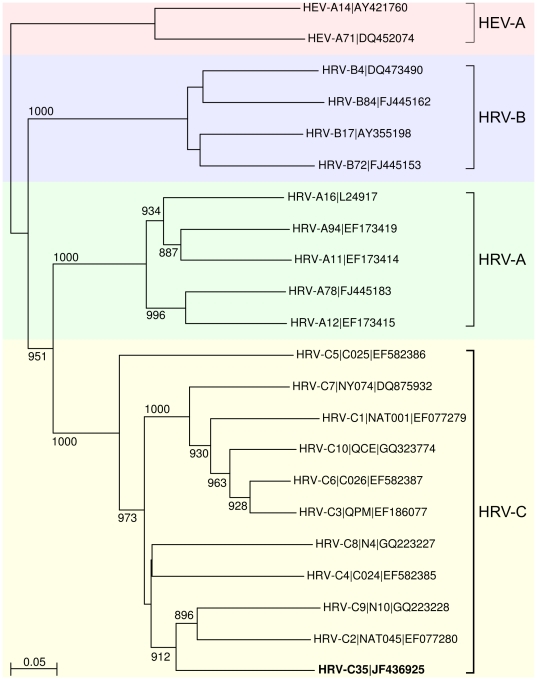
Phylogenetic analysis of the HRV-C35 contigs. Phylogram, based on the VP1 region, showing genetic relationships between the new HRV-C35 (bottom, highlighted) with other publically available HRV-C. Some representative HRV-A, HRV-B serotypes were included in the analysis and two HEV-A serotype sequences were used as outgroup. The neighbor-joining tree was evaluated by 1,000 bootstrap pseudoreplicates. Only bootstrap values over 70% are shown. The distinct clusters representing distinct types are indicated by vertical bars. The scale bar represents the genetic distance.

Among the rhinovirus C sequences, two long contigs, spanning the whole VP1 gene, could be assembled. Both contigs shared less than 80% amino acid identity to known rhinoviruses, which likely suggests the presence of novel types of rhinovirus C in our samples. Human Rhinovirus C (HRV-C) of the Enterovirus genus is a recently discovered species that is associated with severe respiratory infections [19]. The first of the two sequences consisted of 6,858 bp in 7,671 reads thus spanning almost the entire genome. Following the guidelines recently proposed as demarcation criteria for novel types of the HRV-C species [20], [21] this genome was deposited into Genbank under the accession number JF436925 and reported to the *Picornaviridae* Study Group (2010-10-19). The study group has tentatively designated the provided sequence as the prototype sequence of a novel type, HRV-C35. The phylogenetic relationship between HRV-C35, other reported members of the HRV-C and representative types of the HRV-A and –B species is shown in Figure 5. The second HRV-C sequence which also covered almost the entire genome (6,482 bp in 898 reads) was identical to HRV-C34 (unpublished results), which we previously extracted from these patient samples. The metagenomic sequence showed two nucleotide substitutions (A to C and W to A) when aligned to the Sanger sequenced and PCR verified sequence previously extracted and submitted as HRV-C34.

#### Orthomyxoviridae

The third most abundant family was Orthomyxoviridae, which accounted for 21% of the viral sequences of the sample pool (Figure 4A). The majority (96%) of contigs from this family were homologous to the influenza A virus, and the remaining contigs belonged to influenza B (2.3%) and to influenza C (1.7%) (Figure S1). All of the influenza A homologs, except two, were most closely related to the H3N2 subtype, while the two exceptions were most closely related to H9N2. For these two exceptions; the difference in identity between the H9N2 and H3N2 database sequences was small. In context of the vast amounts of H3N2 in the samples and since H9N2 is a bird strain of influenza [22], although human infections have been described [23], it is more likely that these two sequences were in fact of H3N2 origin. H3N2 was the predominant influenza A subtype circulating in Stockholm in 2004 and 2005.

### Other virus findings

In addition to the more prevalent viruses, we also observed a multitude of less well-represented viruses, ranging from known pathogens with/without confirmed human respiratory tract pathogenicity to likely environmental contaminants. The results are summarized in Figure 4A and Table 3 and we will highlight some of the findings here.

#### Human bocavirus (Parvoviridae)

The sample contained 161 contigs that were homologous to Parvoviridae and the vast majority (154) were close homologs to human bocavirus. Among these, all but two contigs shared the highest nucleotide identity with two previously described strains, st1 and st2 [5] (DQ000495, DQ000496). The samples from which human bocavirus was originally discovered were included in the studied sample pools [5]. Thus, st1 and st2 were likely to be the dominant bocavirus sequences in the DNA library. The two longest human bocavirus contigs were both homologous to st2 (in total 3,860 reads) and together they covered almost the entire st2 genome. Due to the high degree of similarity between the st1 and the st2 isolates it was impossible to reliably assign the shorter contigs to either of the two strains.

#### Polyomavirus (Polyomaviridae)

12 contigs of 25 reads were identified as KI polyomavirus, a human polyomavirus that was originally discovered from a sample included in the pool [16]. The low number of reads suggested a very low genome copy number in the original samples. There is no evidence indicating that KI polyomavirus is a respiratory pathogen. It appears that various human polyomaviruses appear at low copy numbers in the respiratory tract, although their pathogenic role may occur in other organs [24].

#### Torque teno virus (Anelloviridae)

The torque teno virus (TTV) was first discovered in a search for potential causative agents of non-A to G hepatitis [25]. TTV have since proven to be an entire family of viruses with remarkable sequence heterogeneity [26], which also includes short anelloviruses called torque teno mini virus (TTMV). TTV and related anelloviruses have been studied extensively, but they have not been shown to be pathogenic [27]. TTV can be detected in >90% of healthy adults [28]. The sequence identities for TTV/TTMV (torque teno mini virus) homologs in our sample ranged down to 35% amino acid identity (for local alignments covering more than 100 bp). This indicates that the samples could possibly contain even more distant anelloviruses that were not detected by BLAST.

#### Human picobirnavirus (Picobirnaviridae)

The RNA-derived library contained a single contig with 70% amino acid identity to the RNA-dependent RNA polymerase of human picobirnavirus. It could be amplified directly from one nasopharyngeal aspirate sample, which confirmed that it originated from a human sample. However, the copy number appeared to be low and the sequence could not immediately be extended further. Human picobirnavirus is the common designation of a range of variable double-stranded RNA-viruses frequently found in human feces [29]. Human picobirnaviruses are poorly studied, and nothing is known about their pathogenicity. It is not even clear whether humans or intestinal microorganisms are the hosts for these viruses. The sequence we recovered was relatively distant from previously reported picobirnavirus sequences, and its classification as a virus is not completely certain.

#### Bell pepper virus (Endornaviridae)

A single contig nearly identical to Bell Pepper Virus (252/253 identical nucleotides, DQ242514) was found. Previously, the occurrence of Pepper Mild Mottle Virus has been described as an indicator of fecal pollution of water as the virus is ingested with food and passes through the gastro-intestinal tract of humans [30]. It is thus likely that in this case, the virus may have contaminated the nasopharynx of a sampled patient.

#### Nanoviridae-like virus

A single read of 252 bp showed weak similarity to the replication initiation protein of banana bunchy top virus (AAG44003). The two sequences were 45.2% identical throughout the amino acid alignment with an e-value of 5e-10. In comparison, the lowest e-value obtained when excluding viral hits was 7e-04. Nanoviridae are currently only known to infect plants. However, sequence homology between Nanoviridae and porcine circovirus suggests that porcine circovirus is distantly related to nanovirus [31] and we cannot exclude the possibility that this fragment could originate from a mammalian virus.

#### Densovirus-like and circovirus-like contigs (Parvoviridae, Circoviridae)

Four contigs from the Parvoviridae family were homologous to various species of the subfamily densovirus, and likely represent a hitherto undescribed densovirus species. Densovirus species are known to infect arthropods. Presence of the densovirus-like sequences was linked to the use of a specific DNA extraction kit (QIAamp DNA Blood Mini Kit, Qiagen). The densovirus-like sequences were found in all samples, including water, when extracted by this kit, but not when extracted by other kits. It was concluded that the densovirus-like sequences were not present in the samples, but was reagent-derived.

The sample contained 11 contigs where the closest homolog was found within the Circoviridae family, with the amino acid identity ranging from 30% up to 78% (for local alignments covering at least 100 bp). For many of these contigs the closest homologs were circovirus-like genomes that were recently found in reclaimed wastewater [32]. The sequence diversity indicates that the contigs may be derived from more than one circovirus species. One of these contigs was further investigated through PCR of original samples. The circovirus-like sequence was not repeatedly amplifiable from any of the original samples, but gave intermittently positive PCR results. This was interpreted as low copy number presence of the circovirus-like sequence. The experience from the densovirus-like sequence prompted an investigation of different extraction methods. Intermittent PCR positivity appeared only when QIAamp DNA Blood Mini Kit (Qiagen) was used. We concluded that this sequence was likely reagent-derived and did not pursue any further investigations.

## Discussion

We have conducted a metagenomic analysis of both the DNA and RNA viromes in patients with severe lower respiratory tract infections. Approximately 700,000 sequence reads were produced using 454 sequencing, corresponding to 110 Mbp when combining the RNA and DNA sample. The samples showed a great diversity in the viral flora, with a total of 4,757 contigs originating from 39 species and an even larger number of strains. We have previously shown that this method is sensitive and thus has high potential for virus identification, even when a small number of sequences were produced [5]. It is clear that the sensitivity of the current protocol is greatly increased, thanks to the capacity of 454 sequencing. While it is possible that a viral genome can go undetected, it is likely that most viruses in the samples are represented in the sequence data, even those with low titer. However, we can not conclude that viruses were not excluded prior to sequencing, for example by filtering steps in the viral enrichment protocol.

We found that by reducing our datasets by removing repetitive reads and reads of human origin pre-assembly, the total time required for sequence assembly and analysis could be significantly reduced and the complexity of the assembly was decreased. This also reduced the risk of mis-assemblies [33] and in our case most notably the risk of chimeric contigs consisting of reads of both human and viral origin. The filtering criteria were set so that a very high degree of homology to repeats and human sequences was required for removal, in order to avoid the loss of sequences of interest.

Viral metagenomics assembly is a non-trivial computational task, even after pre-assembly screening, and there are no specific metagenomic assembly programs. However, most genome assemblers appear to solve metagenomic assemblies relatively well [34]. We have used the MIRA program [12], since Newbler [35] incorrectly tagged clearly non-repetitive viral sequences as repetitive, most likely due to the variation in coverage caused by uneven titers of the viruses in the original samples.

We used a more complex method for classification of sequences than in previous studies, where native BLASTx homolog classification was used for the most part. We improved on this by using thorough nucleotide and translated nucleotide comparison in combination. Also, an additional comparison step was added after BLAST hits were identified in order to improve the classification. In this step, the scores of the viral hits were compared with the scores of the non-viral hits. As an example, this measurement could reveal the difference between a distinctly viral RNA-dependent RNA polymerase (RNAP) and an RNAP that also shared high identity to a bacterial RNAP. Viral sequences that were not sufficiently distinct from other categories, most commonly bacteria, could therefore be given lower priority.

The vast majority of viruses identified in this study belonged to three abundant families that were dominated by four virus species, namely *Paramyxoviridae* (hRSV and hPIV), *Orthomyxoviridae* (influenza virus) and *Picornaviridae* (HRV), all known to be present in the human respiratory tract. We also found other viruses known to be replicating in these tissues; including human bocavirus, human coronavirus and measles virus as well as confirmed human viruses for which no pathogenicity has been described, such as TTV and KI polyomavirus. Thus, regarding previously known viruses, the results of this study confirmed previous studies of human respiratory tract viruses [3], [6], [15], [17]. In addition, our results expanded the number of identified strains and possibly types and species of anellovirus and rhinovirus. In particular, we identified one likely new type of human rhinovirus C designated by the Study Group of Picornaviruses as the prototype of human rhinovirus C35. While a vast multitude of viruses was found we cannot exclude that the viral enrichment protocol may exclude some, particularly large, viruses which may be relevant human pathogens.

A large proportion of the sequences from viruses and bacteria have been confirmed to be a part of the human microflora and many were in fact from known pathogens of the nasopharyngeal tract. However, while the sampled population suffered from severe lower respiratory tract infections, the nasopharyngeal aspirates collected are likely to contain virus which mainly replicate in the upper respiratory tract. The mucosa may also contain temporary microorganisms that are not part of the normal microflora. They may come from the environment, for example from dust and from food and water. It is likely that a proportion of the sequences came from such microorganisms. In addition, the reagents used for sample processing may contribute both viral and bacterial sequences. Furthermore, as no healthy controls were sampled the significance of any findings as causing lower respiratory tract infections, can not be established.

We have attempted to provide a complete picture of the viral content of these samples and have applied stringent quality filters to ensure correct classification of the metagenomic data. Even so, incomplete databases made accurate classification problematic in some cases where no, or only very distant, homologs were found. We anticipate that this problem will decrease as the public databases grow. A related problem was low scores caused by short sequences. Some of our virus findings will need to be confirmed by additional sequencing to provide the remainder of the virus genomes. We have also provided a brief overview of the bacterial contigs where we found several pathogens. While these results confirm previous finding [15], due to the viral purification performed we cannot provide an unbiased characterization of the bacterial content in these patients.

It is clear that viral metagenomics provides a crucial tool for virus discovery. Using this approach; no *a priori* information is needed, as in directed PCR assays, and viruses that are difficult to propagate in cell culture can be discovered. Furthermore, our reconfirmation of the sequence of HRV-C34 show remarkable accuracy of the metagenomic assembly and ability to recover a HRV-C isolate in the presence of other isolates. Our results highlight the strength of the method to not only identify novel viruses, but also to identify viruses that were likely to be missed by ordinary clinical tests. As sequencing continuously becomes more available and inexpensive, it could also become a viable clinical diagnostic method.

## Materials and Methods

A schematic overview of the entire process from sample collection and preparation throughout the data-analysis is given by Figure 1. The first two steps describe sample collection up to sequencing and the following steps (3 to 6) describe *in silico* analyzes.

### Sample collection

All patient samples were analyzed anonymously and the study was approved by the local ethics committee. Two hundred and ten randomly selected, anonymized, nasopharyngeal aspirates were included in the study. The samples were originally submitted to the Karolinska University Laboratory, Stockholm, Sweden from March 2004 to May 2005 for diagnosis of respiratory tract infections. The majority of samples were from the children's hospital at Karolinska. 70% of the samples were derived from children under 7 years. The remaining 30% of samples were mainly from adults (mean age 44 years, range 8–92), and collected for diagnosis of suspected influenza. The symptoms of the individual patients were not recorded due to the study design with anonymized samples. However, the general policy of the children's hospital is to sample only inpatients and not outpatients for respiratory viruses. It is therefore reasonable to assume that the majority of samples are from patients with symptoms severe enough to require hospitalization. The most common symptoms reported in children hospitalized for respiratory tract infections are fever, cough, and wheezing.

### Ethics statement

The project is based on analysis of human clinical samples. In order to avoid ethical complications, samples were anonymized and cannot be traced back to individual patients. The study was approved by the local ethics committee at the Karolinska Institute, The Regional Ethical Review Board, Stockholm, Dnr 02-212, 02-422, and 04-836/4. Since the samples are completely anonymous, the ethical board determined that no informed consent from the patients was needed.

### Library construction & sequencing

The samples were processed in 13 pools with 8–24 samples each, using our previously published protocol [5], [16]. In brief, samples were pooled and each pool was divided into two aliquots, which were filtered through 0.22- and 0.45-µm-pore-size disc filters (Millex GV/HV; Millipore), respectively. Both aliquots were ultracentrifuged at 41,000 rpm in an SW41 rotor (Beckman) for 90 min. The resulting pellet was recovered, resuspended, and treated with DNase before DNA and RNA were extracted [36]. Extracted DNA and RNA were amplified separately by “random PCR” [5], [37]. The primer sequences were then removed from the amplification products by restriction enzyme digest before the amplification products were separated on an agarose gel, and fragments between approximately 400 and 1,500 bp in length were cut out and purified for use as sequencing template. This resulted in a total of 13 DNA libraries and 13 cDNA libraries, which were in turn pooled to two libraries representing the DNA content and the RNA content of the sample, respectively. The DNA and RNA-derived libraries were sequenced separately, using the 454 sequencing platform [35]. The first sequencing run was performed on a GS20 instrument and the second sequencing run was performed on the enhanced GS FLX instrument. Both runs were of two plates each, one DNA and one RNA plate. The sequencing produced in total 703,790 reads, the runs are summarized in Table S1. This Whole Genome Shotgun project has been deposited at DDBJ/EMBL/GenBank under the accession AFAP00000000. The version described in this paper is the first version, AFAP01000000. In addition, two new Rhinovirus C variants have been deposited, accession numbers JF436925–JF436926.

### In silico analysis

Initial studies of the samples suggested that the RNA and DNA sequencing runs shared some content. Due to this overlap in sample content it was beneficial to analyze the 454 sequencing runs together, rather than as separate RNA and DNA analyzes. By doing so, longer contigs could be formed by combining the reads from the two pools but it was still possible to deduce the origin of each read from the assembly. For example, each assembled contig was tagged with origin such as DNA/RNA or a mixture of both. These *in silico* steps are shown in Figure 1 as ‘Step 3’ through ‘Step 6’.

#### Pre-assembly screening for low complexity and human content

The pre-assembly screening was performed using RepeatMasker [38] and NCBI BLAST [14]. The screening process was performed in three steps where at each step any read which did not fulfill a pass criterion was discarded. The first step involved running the reads through RepeatMasker to produce masked FASTA files used for further analyzes. Reads were discarded if more than 70% of the nucleotides were masked, or if highest scoring stretch (given a 1/-5 model for non-N and N respectively) was shorter than 50 bp. The 50 bp threshold was chosen as a cut-off as shorter sequences were both rare and provided little information. The thresholds for the repetitive classification model were determined empirically with the goal of purging only heavily repetitive sequences. The next two steps of the screening process were performed using NCBI BLAST; searching against first the NCBI databases Human Genome Transcripts and then the Human Genome database [13]. At each consecutive step further reads were discarded if a homolog was found at or above 90% identity covering at least 80% of the query sequence. These thresholds were set after manual inspection of the sequence identity and coverage range, with the goal of removing reads that would after a full pipeline run still be classified as of human origin.

#### De novo genome assembly

Sequence assembly was performed using the MIRA 3.0.5 software [12] with the parameters ‘-job = denovo,genome,accurate,454’. The resulting ACE-file was further analyzed and contig statistics were extracted for each contig. The following information was extracted: number of reads, contig coverage (min, max and mean) as well as sample origin (DNA or RNA pool). The final result of this process was regular FASTA output with all extracted information added to each FASTA header line.

#### Homolog searches

The homolog searches conceptually consisted of searching against both a nucleotide and protein database, translating the nucleotide sequences in all six possible frames, see ‘Step 5’ of Figure 1. The similarity search was partitioned into three levels of homology-search, where at each level; any sequence that could be reliably classified was removed from downstream analyzes. For nucleotide homology search, the NCBI nt (minimally non-redundant nucleotide) database (Jan 2010) [13] was used and for the six-frame translated nucleotide homology search the NCBI nr (non-redundant protein) database (Jan 2010) [13] was used.

At the first level, the search pipeline consisted of a nucleotide BLAST search using the MegaBLAST algorithm [39] with parameter settings optimized for finding homology around 90% identity (2/-3 reward/penalty and 5/2 gap open/extend cost). Sequences for which a homolog were found with at least 90% identity covering 70% or more of the query sequence, were dismissed from further search, at each level. The second level of homology searches was performed using the BLASTn algorithm with parameter settings optimized for finding homologs of lower identity (4/-5 reward/penalty and 12/8 gap open/extend cost). The final search step was performed using the BLASTx algorithm using default parameters. All BLAST searches were performed using the default e-value cut-off of 10, while more stringent thresholds were employed in downstream analysis.

For each supplied query sequence, the BLAST program returns a pre-defined number (*n*) of likely homologs in descending order. Thus, potentially interesting homologs that were not within these *n* most likely homologs were not included in the result. For example, a bacterial RNA-polymerase homolog might not be within these *n* most likely homologs if there were *n* better (often very similar) viral homologs in the database. This complicates query analysis and to address this problem, each search step was partitioned into searches against four distinct subsets consisting of mammalian sequences, bacterial sequences, viral sequences and ‘all other’ sequences. These four subsets will here be denoted ‘categories’. By dividing the database into these categories the highest scoring homologs of each group were identified, regardless of the score of homologs within other categories. For each query, at each level of database searches, the top-three hits of each category (mammals, bacteria, viruses and others) were kept. The hits were then ranked using bit-score instead of e-value, in order to avoid the database size bias introduced into the e-value.

#### Comparative analysis of homologs

Chimeric reads may result from unspecific PCR reactions and such reads may in turn cause mis-assembled contigs. Therefore, upon completion of the second level of databases search (thorough search against NT using BLASTn) the hits of each query were analyzed. A list of equal length to the query was created for each category (mammals, bacteria, viruses and others) where the highest bit-score covering each position was noted, called a score map. These score maps for each category were analyzed to allow splitting a sequence if two or more parts of the sequence had their closest homolog within different categories. The algorithm consisted of four major steps:

1. First, parts of the query sequence were identified as a continuous sub-sequence of the query, longer than 50 bp, for which the highest scoring (bit-score) homolog in each position were part of the same particular category.
2. These parts were then analyzed and if any two parts were found matching different categories; a potential split (in between the relevant two ‘parts’) of the sequence was evaluated.
3. The sequence was split into sub-sequences if, for both sub-sequences, the bit-score of the relevant category was on average at least 2 times as high as the bit-score of any other category, covering the same sub-sequence, *i.e.* if the mean bit-score ratios for both subsequences were at least 2.
4. Finally, the resulting sub-sequences were re-queried against the database and the sub-sequences replaced the original sequence in the query-set.

In order to provide a more stable range for these bit-score ratios, calculated in step 3 above, as well as avoiding low scoring alignments from splitting sequences; a maximum ratio of 10.0 as well as a minimum bit-score of 20.0 was allowed for each position. Thus, the top scoring alignment bit-score for a category *n* at position *p* (*s_n,p_*) is normalized to *S_n,p_* = *max*(*s_n,p_*, 20.0). Furthermore, the bit-score ratio for category *n* at position *p*, *r_n,p_* = *S_n,p_*/*max_k≠n_ (S_k,p_)* is normalized to *R_n,p_* = *min*(*r_n,p_*, 10.0). These thresholds were set after manual classification of 15 chimeric sequences so that the pipeline would split all these (using these thresholds identified 90 contigs in total).

After completion of all three levels of homology searches the result-set was compiled into a final result-set. Each sequence was assigned to the category of the highest scoring (bit-score) hit from homology searches if:

1. The e-value of the hit was below or equal to 10^−3^
2. The mean position-wise bit-score ratio between the suggested category and other categories, over the entire sequence was at least 1.15.
3. If the bit-score of the top-scoring hit was at least 33% higher than the second highest scoring hit, the mean position-wise bit-score ratio was ignored thus introducing a top-scoring hit ‘veto’ which would cancel the 2^nd^ requirement.

If these conditions were not met, the particular sequence was instead assigned to the ‘undefined’ category, thus containing sequences for which no close homolog was found or where homologs of seemingly indistinguishable importance suggest different categories. The e-value threshold of 10^−3^ was used as in previous studies [8] while the thresholds for conflicting homology were determined empirically.

Finally, through the use of the NCBI taxonomy database [13] each sequence could be further partitioned at any taxonomic level by considering the closest homolog. All of the categories were first automatically split according to NCBI taxonomy division [13], for example as in Table S2 and Table S3. To gain a more granular grouping; specific parts of the results were further partitioned, for example as in Figure 4 and Figure S1.

#### Phylogenetic analysis of novel HRV-C genome sequence

Multiple sequence alignments and the phylogenetic tree were prepared using ClustalX 2.0.12 [40]. Genetic distances were calculated using the Kimura-2 parameter and a Ts/Tv ratio of 2.0. The phylogenetic tree was constructed using the neighbor-joining method and evaluated by 1,000 bootstrap pseudoreplicates. The tree was ploted using NJplot 2.3 [41].

## Supporting Information

Figure S1 **Species of the Orthomyxoviridae family.** The *Orthomyxoviridae* homolog sequences split by species (manually curated, only alignments with an e-value<1e-5 considered). The numbers are the derived number of reads.
(TIFF)

Table S1 **Sequencing and screen information.** Sequencing runs performed using both the GS20 and the GS FLX 454 sequencing instrument as well as screening efforts removing repetitive sequences and/or sequences of human origin.
(DOC)

Table S2 **Assembly information.** The number or reads, sequences, longest sequences and total bases for assembled sequences (contigs) and singletons. Note that 10,951 of the sequenced reads did not form contigs and were too short to be included as singletons for further analysis (the exclusion process is described further in Materials and Methods).
(DOC)

Table S3 Chromosomal distribution of human contigs. Chromosomal distribution of sequences most homologous to *Homo sapiens* defined by closest homolog.
(DOC)

Table S4 **Taxonomy category break-down of ‘others.’** The contigs of the ‘others’ category defined by closest homolog and split by taxonomy division.
(DOC)

Table S5 **Taxonomy category break-down of ‘undefined.’** The contigs of the ‘undefined’ category defined by closest homolog and split by taxonomy division.
(DOC)
