# Supplementary figures and images for: Characterization of the Viral Microbiome in Patients with Severe Lower Respiratory Tract Infections, Using Metagenomic Sequencing
Source: PLoS One. 2012 Feb 15;7(2):e30875. doi: 10.1371/journal.pone.0030875 (PMC3280267; doi:10.1371/journal.pone.0030875)

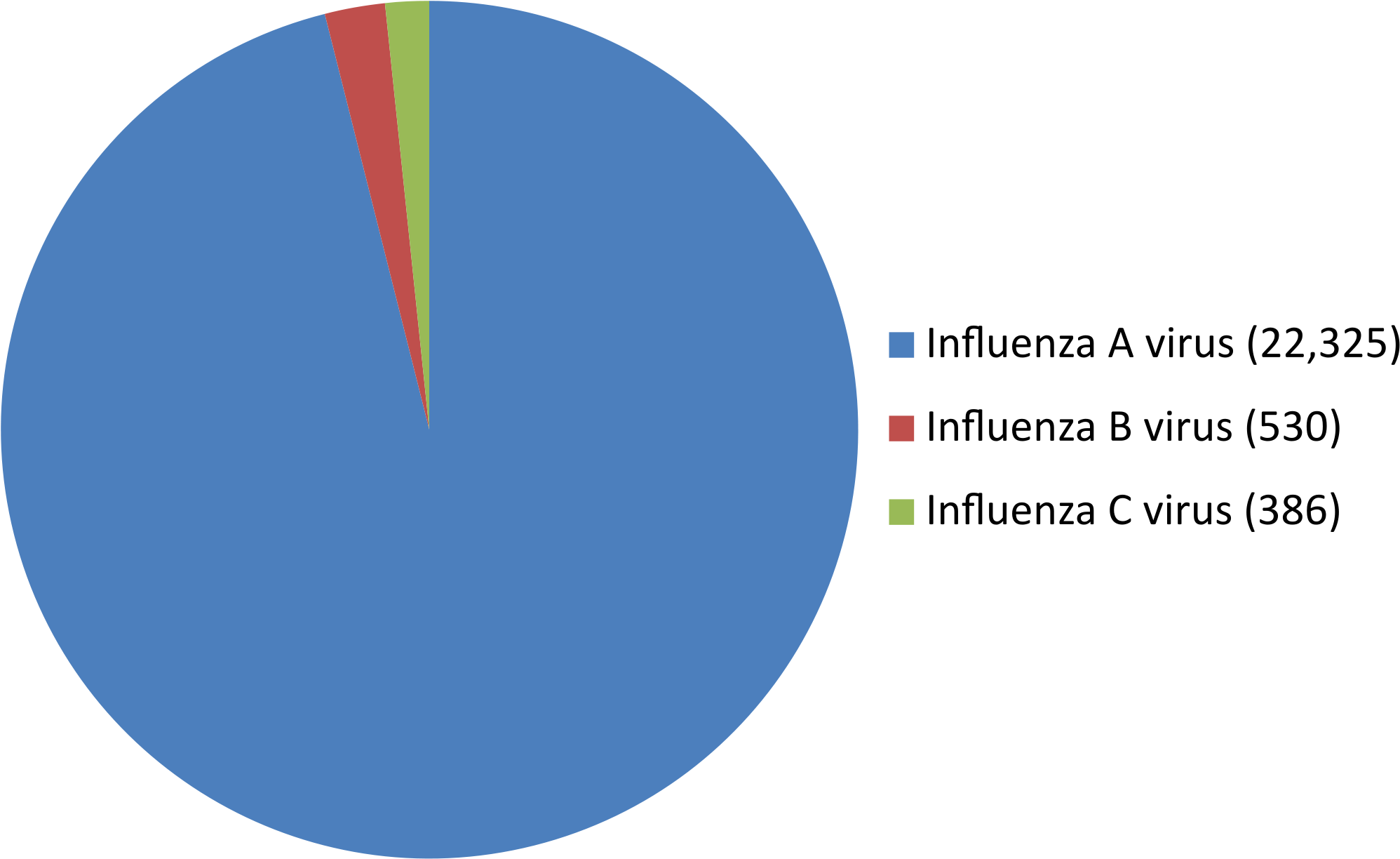

Supplement: Figure S1 — Species of the Orthomyxoviridae family. The Orthomyxoviridae homolog sequences split by species (manually curated, only alignments with an e-value<1e-5 considered). The numbers are the derived number of reads. (TIFF) [file pone.0030875.s001.tiff]
