# Supplementary material for: Characterization of the Viral Microbiome in Patients with Severe Lower Respiratory Tract Infections, Using Metagenomic Sequencing
Source: PLoS One. 2012 Feb 15;7(2):e30875. doi: 10.1371/journal.pone.0030875 (PMC3280267; doi:10.1371/journal.pone.0030875)
Supplement: Table S1 — Sequencing and screen information. Sequencing runs performed using both the GS20 and the GS FLX 454 sequencing instrument as well as screening efforts removing repetitive sequences and/or sequences of human origin. (DOC) [file pone.0030875.s002.doc]

Table S1. Sequencing and screen information.

| **Instrument** | **Type** | **No. of reads** | **Sequenced bases** | **Reads screened** | **Reads remaining** |
| --- | --- | --- | --- | --- | --- |
| GS20 | DNA | 160,120 | 15.95 Mbp | 113,413 | 46,707 (29.17%) |
| GS20 | RNA | 138,941 | 14.27 Mbp | 66,106 | 72,835 (52.42%) |
| GS FLX | DNA | 165,715 | 30.81 Mbp | 102,382 | 63,333 (38.22%) |
| GS FLX | RNA | 239,014 | 49.73 Mbp | 125,242 | 113,772 (47.60%) |
| **Total** |  | **703,790** | **110.77 Mbp** | **407,143** | **296,647 (42.15%)** |

Sequencing runs performed using both the GS20 and the GS FLX 454 sequencing instrument as well as screening efforts removing repetitive sequences and/or sequences of human origin.
