# Supplementary material for: Characterization of the Viral Microbiome in Patients with Severe Lower Respiratory Tract Infections, Using Metagenomic Sequencing
Source: PLoS One. 2012 Feb 15;7(2):e30875. doi: 10.1371/journal.pone.0030875 (PMC3280267; doi:10.1371/journal.pone.0030875)
Supplement: Table S2 — Assembly information. The number or reads, sequences, longest sequences and total bases for assembled sequences (contigs) and singletons. Note that 10,951 of the sequenced reads did not form contigs and were too short to be included as singletons for further analysis (the exclusion process is described further in Materials and Methods). (DOC) [file pone.0030875.s003.doc]

Table S2. Assembly information.

|  | **No. of reads** | **No. Sequences** | **Max length** | **Total bases** |
| --- | --- | --- | --- | --- |
| **Contigs** | 185,506 | 9,300 | 3,746 bp | 2.34 Mbp |
| **Singletons** | 100,190 | 100,190 | 402 bp | 16.0 Mbp |
| **Total** | **285,696** | **109,490** | **3,746 bp** | **18.34 Mbp** |

The number or reads, sequences, longest sequences and total bases for assembled sequences (contigs) and singletons. Note that 10,951 of the sequenced reads did not form contigs and were too short to be included as singletons for further analysis (the exclusion process is described further in Materials and Methods).
