# Supplementary material for: Characterization of the Viral Microbiome in Patients with Severe Lower Respiratory Tract Infections, Using Metagenomic Sequencing
Source: PLoS One. 2012 Feb 15;7(2):e30875. doi: 10.1371/journal.pone.0030875 (PMC3280267; doi:10.1371/journal.pone.0030875)
Supplement: Table S3 — Chromosomal distribution of human contigs. Chromosomal distribution of sequences most homologous to Homo sapiens defined by closest homolog. (DOC) [file pone.0030875.s004.doc]

Table S3. Chromosomal distribution of human contigs.

|  | **Contigs** | **Contigs per Mbp** |
| --- | --- | --- |
| **Chromosome 1** | 3,862 | 17.2 |
| **Chromosome 2** | 3,515 | 14.8 |
| **Chromosome 3** | 2,692 | 13.8 |
| **Chromosome 4** | 2,484 | 13.3 |
| **Chromosome 5** | 2,545 | 14.3 |
| **Chromosome 6** | 2,398 | 14.3 |
| **Chromosome 7** | 2,338 | 15.1 |
| **Chromosome 8** | 2,137 | 15.0 |
| **Chromosome 9** | 1,930 | 16.0 |
| **Chromosome 10** | 2,051 | 15.6 |
| **Chromosome 11** | 2,266 | 17.3 |
| **Chromosome 12** | 2,011 | 15.4 |
| **Chromosome 13** | 1,265 | 13.2 |
| **Chromosome 14** | 1,219 | 13.8 |
| **Chromosome 15** | 1,254 | 15.4 |
| **Chromosome 16** | 1,382 | 17.5 |
| **Chromosome 17** | 1,337 | 17.2 |
| **Chromosome 18** | 1,119 | 15.0 |
| **Chromosome 19** | 1,190 | 21.3 |
| **Chromosome 20** | 1,152 | 19.4 |
| **Chromosome 21** | 588 | 17.2 |
| **Chromosome 22** | 693 | 19.9 |
| **Chromosome X** | 1,325 | 8.8 |
| **Chromosome Y** | 242 | 9.6 |
| **Mitochondrion** | 901 | 54,000 |
| **Undefined/Repetitive** | 6,840 | - |

Chromosomal distribution of sequences most homologous to *Homo sapiens* defined by closest homolog.
