# Supplementary material for: Characterization of the Viral Microbiome in Patients with Severe Lower Respiratory Tract Infections, Using Metagenomic Sequencing
Source: PLoS One. 2012 Feb 15;7(2):e30875. doi: 10.1371/journal.pone.0030875 (PMC3280267; doi:10.1371/journal.pone.0030875)
Supplement: Table S4 — Taxonomy category break-down of ‘others.’ The contigs of the ‘others’ category defined by closest homolog and split by taxonomy division. (DOC) [file pone.0030875.s005.doc]

Table S4. Taxonomy category break-down of ‘others’.

| **Taxonomy division** | **Reads** |
| --- | --- |
| Plants | 1,508 |
| Invertebrates | 1,320 |
| Vertebrates | 725 |
| Phages | 557 |
| Environmental samples | 54 |

The contigs of the ‘others’ category defined by closest homolog and split by taxonomy division.
