# Supplementary material for: Characterization of the Viral Microbiome in Patients with Severe Lower Respiratory Tract Infections, Using Metagenomic Sequencing
Source: PLoS One. 2012 Feb 15;7(2):e30875. doi: 10.1371/journal.pone.0030875 (PMC3280267; doi:10.1371/journal.pone.0030875)
Supplement: Table S5 — Taxonomy category break-down of ‘undefined.’ The contigs of the ‘undefined’ category defined by closest homolog and split by taxonomy division. (DOC) [file pone.0030875.s006.doc]

Table S5. Taxonomy category break-down of ‘undefined’.

| **Taxonomy division** | **Reads** |
| --- | --- |
| No homology (e-value above 1e-3) | 23,974 |
| Environmental samples | 4,316 |
| Primates | 2,727 |
| Bacteria | 1,448 |
| Synthetic | 897 |
| Plants | 506 |
| Invertebrates | 424 |
| Rodents | 322 |
| Vertebrates | 257 |
| Viruses | 122 |
| Mammals | 74 |
| Phages | 46 |

The contigs of the ‘undefined’ category defined by closest homolog and split by taxonomy division.
